# Unveiling the aesthetic secrets: exploring connections between genetic makeup, chemical, and environmental factors for enhancing/improving the color and fragrance/aroma of *Chimonanthus praecox*

**DOI:** 10.7717/peerj.17238

**Published:** 2024-04-19

**Authors:** Haoyu Zhao, Hafiza Ayesha Masood, Sher Muhammad

**Affiliations:** 1 MEU Research Unit, Middle East University, Amman, Jordan; 2 Faculty of Social and Cultural Communications, Belarusian State University, Minsk, Belarus; 3 Department of Biotechnology, University of Okara, Okara, Punjab, Pakistan

**Keywords:** *Chimonanthus praecox*, Flower color, Flower fragrance, Genetic regulation, Landscape design

## Abstract

Floral color and scent profiles vary across species, geographical locations, and developmental stages. The exclusive floral color and fragrance of *Chimonanthus praecox* is contributed by a range of endogenous chemicals that distinguish it from other flowers and present amazing ornamental value. This comprehensive review explores the intricate interplay of environmental factors, chemicals and genes shaping the flower color and fragrance of *Chimonanthus praecox*. Genetic and physiological factors control morpho-anatomical attributes as well as pigment synthesis, while environmental factors such as temperature, light intensity, and soil composition influence flower characteristics. Specific genes control pigment synthesis, and environmental factors such as temperature, light intensity, and soil composition influence flower characteristics. Physiological processes including plant hormone contribute to flower color and fragrance. Hormones, notably ethylene, exert a profound influence on varioustraits. Pigment investigations have spotlighted specific flavonoids, including kaempferol 3-O-rutinoside, quercetin, and rutin. Red tepals exhibit unique composition with cyanidin-3-O-rutinoside and cyanidin-3-O-glucoside being distinctive components. Elucidating the molecular basis of tepal color variation, particularly in red and yellow varieties, involves the identification of crucial regulatory genes. In conclusion, this review unravels the mysteries of *Chimonanthus praecox*, providing a holistic understanding of its flower color and fragrance for landscape applications. This comprehensive review uniquely explores the genetic intricacies, chemical and environmental influences that govern the mesmerizing flower color and fragrance of *Chimonanthus praecox*, providing valuable insights for its landscape applications. This review article is designed for a diverse audience, including plant geneticists, horticulturists, environmental scientists, urban planners, and students, offering understandings into the genetic intricacies, ecological significance, and practical applications of *Chimonanthus praecox* across various disciplines. Its appeal extends to professionals and enthusiasts interested in plant biology, conservation, and industries dependent on unique floral characteristics.

## Introduction

The term ‘ornamental plant’ encompasses all plants appreciated for their aesthetic appeal, including beautiful flowers or distinctive architectural features, boasts a wealth of ornamental plant resources that play a crucial role in enhancing living environments, cultivating human sentiment, and contributing to structural adjustments within the agricultural industry (Zheng et al., 2021; Francini et al., 2022). One noteworthy ornamental plant is the wintersweet (*Chimonanthus praecox* (L.) Link), which has developed distinctive fragrant aroma and winter-flowering properties critical for its effective sexual reproduction (Shang et al., 2020). Currently, wintersweet holds commercial importance as a potted and landscape plant, gaining popularity globally. Due to its exceptional flowering time and distinctive fragrance during the winter months, wintersweet has been introduced to various regions worldwide, including Japan, Korea, Europe, Australia, and America and is highly prized in temperate China (Sui et al., 2015; Hu et al., 2020; Huang et al., 2021). Wintersweet exhibits a primary distribution in the southern, central, eastern, southwestern, and northwestern regions of China (Chen, 2012), and has a rich history of cultivation in China, spanning over a millennium (Zhao et al., 2007; Xiang, Zhao & Chen, 2010).

Wintersweet flowers find diverse uses, from potpourri and linen scenting to essential oil extraction for cosmetics, perfumes, and aromatherapy. Additionally, wintersweet flowers are employed in herbal teas and various Chinese folk remedies for ailments ranging from coughs to measles (Lv et al., 2012). Ongoing research by various researchers focuses on uncovering the health benefits of wintersweet, attributed to its antifungal, antioxidant, and biocidal properties (Lv et al., 2012). Its popularity is attributed to its unique characteristics, including a winter blossoming period, exciting yellow blossoms, and a potent fragrance, establishing it as a favored ornamental plant (Zhao et al., 2007). The distinctive fragrance of *Chimonanthus praecox* has led to its widespread use in landscaping, as cut flowers, and as a material for bonsai (Lin & Chen, 2020; Meng et al., 2021). Beyond ornamental purposes, this plant holds economic significance in industries such as tea production, essential oils, and cosmetics (Lu et al., 2020). Additionally, *Chimonanthus praecox* is recognized for its rich content of volatile components, presenting considerable potential in drug research and development (Li, 2018; Xu et al., 2018; Shen et al., 2020).

The distinctive floral fragrance of *Chimonanthus praecox* originates from volatile compounds and fulfills diverse ecological and economic functions. These encompass deterring pathogens (Arimura et al., 2004) and herbivores (Pichersky & Gershenzon, 2002; Unsicker, Kunert & Gershenzon, 2009), safeguarding flowers against detrimental insects (Li et al., 2017), enticing pollinators (Dudareva, Pichersky & Gershenzon, 2004), fostering medicinal attributes (Zhao et al., 2012), enabling plant-to-plant communication (Raguso, 2008; Schiestl, 2010), elevating aesthetic appeal, and even drawing tourists (de Vega, Herrera & Dötterl, 2014), among others. These manifold roles underscore the significance of floral fragrances in both scientific research and economic considerations.

Flowering, a pivotal stage in the plant life cycle, is intricately regulated by both internal indications and a myriad of environmental factors (Noman et al., 2017; Cho, Yoon & An, 2017). Various tree species exhibit distinct seasonal patterns in their flowering. Wintersweet, characterized by its winter blooming, is particularly notable for its flowers, which emit an intense fragrance attributed to a mixture of volatile terpenoids (sesquiterpenes and monoterpenes) and benzenoids (Tian et al., 2019). These fragrant compounds are released from nectaries located on the adaxial surface of inner petals (Li et al., 2018). The essential oils derived from wintersweet flowers find widespread applications in the perfume, cosmetics, and flavor industries, owing to their unique and pleasant aroma (Deng, Song & Hu, 2004; Azuma, Totota & Asakawa, 2005). Beyond their industrial use, the floral scent serves crucial ecological functions. It plays a pivotal role in attracting and guiding pollinators, ensuring the plant’s reproductive success. Additionally, the fragrance acts as a defense mechanism, protecting the plant’s susceptible reproductive structures from pathogens and florivores (Huang et al., 2012).

**Figure 1:**
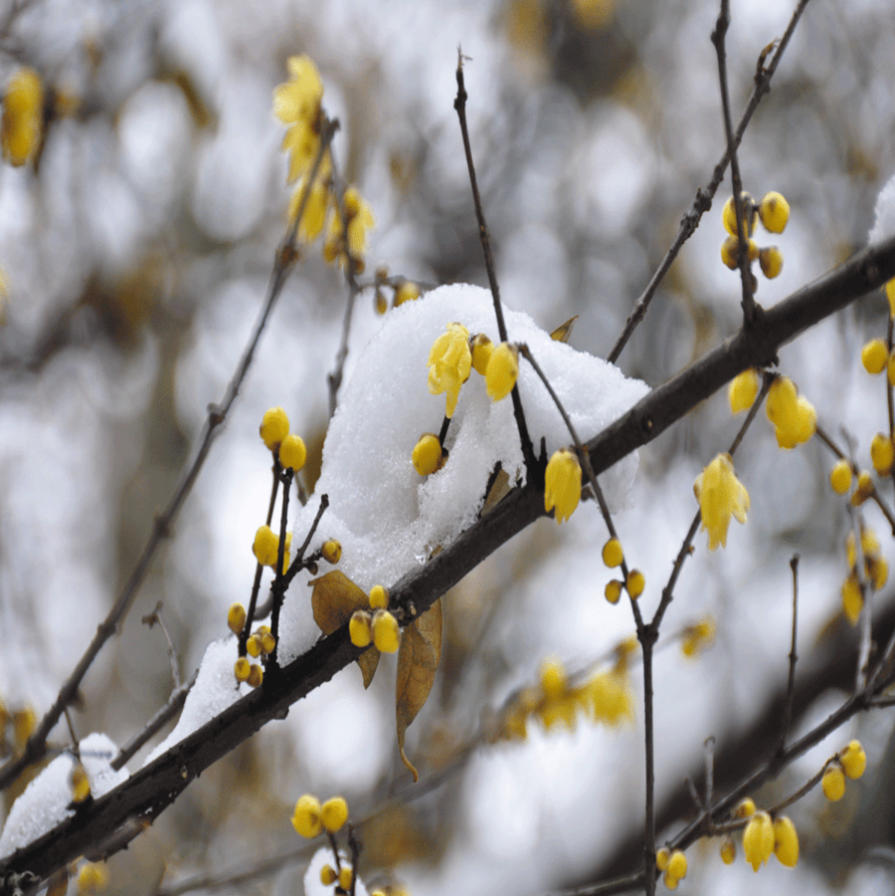
Wintersweet flower morphology, blooming in winter.

Wintersweet has developed a remarkable adaptation to coordinate its blossoming with seasonal climate fluctuations, particularly responding to temperature variations. The process of flower origination begins in spring, yet the actual blooming of flowers occurs throughout winter, normally in late December or early January (Shang et al., 2020). Throughout the summer and autumn months, flower buds exhibit notably slow growth, undergoing the intricate stages of floral organ specification, maturation, and differentiation (Bartolini, Piccolo & Remorini, 2020). In preparation for the impending winter, wintersweet, like many long-lived trees, experiences development termination and enters a state of dormancy (Shen et al., 2021). What distinguishes wintersweet from the majority of flowering trees is its unique flowering season, wherein floral buds emerge and flowers bloom during the winter months. This characteristic is visually depicted in Fig. 1, sourced from Shang et al. (2020). This unusual blooming period necessitates the development of robust cold hardiness in wintersweet flowers. Furthermore, wintersweet flowers display a unique feature in having a fully petaloid perianth without differentiation of petals and sepals (Kubitzki, 1993). These distinctive characteristics make wintersweet an intriguing system for studying various aspects of plant biology, including the regulation of flower development, flowering time, fluorescent bud dormancy, and bud break (Shang et al., 2020).

This review aims to investigate the genetic basis of flower color variation in *Chimonanthus praecox*, focusing on key genes and molecular pathways regulating pigment biosynthesis and to examine the environmental influences on floral traits, elucidating how factors like temperature, light, and soil composition contribute to variations in flower color and fragrance. Moreover, this review assess the practical applications of understanding Chimonanthus praecox genetics and ecology in landscape design, providing insights for horticulturists to create aesthetically pleasing and ecologically sustainable outdoor environments.

### Survey/search methodology

To ensure comprehensive and unbiased coverage, a systematic search strategy incorporating diverse databases such as Scopus, Web of Science, PubMed, and Google Scholar, peer-reviewed journals, and reputable sources was employed.

### Inclusion and exclusion criteria

Inclusion and exclusion criteria were clearly defined, favoring transparency and minimizing publication bias. The iterative review process and adherence to systematic review guidelines further ensured a thorough and impartial exploration of the literature on *Chimonanthus praecox*.

Primary keywords were initially used to identify relevant works and secondary key terms were used to ensure that potentially relevant results were not missed. Conventional search engines such as Google, Bing, and DuckDuckGo were also utilized to ensure recent non-indexed works were also captured.

### Primary key terms included

*Chimonanthus praecox* AND flower color

*Chimonanthus praecox* AND flower fragrance

*Chimonanthus praecox* AND genetic regulation

Environmental influences, flower color AND *Chimonanthus praecox*

*Chimonanthus praecox*, flower color, fragrance AND landscape design

*Chimonanthus praecox*, flower color, fragrance AND ecological significance

## Botanical Overview of *Chimonanthus praecox*

*Chimonanthus praecox*, commonly identified as wintersweet or Japanese allspice, is a deciduous shrub native to China and belongs to the *Chimonanthus* genus within the *Calycanthaceae* family (Paudel & Heo, 2018). In Chinese culture, it is often referred to as the “Plum Blossom”, while in Persian, it is cultivated as “Gorye Yak” in Iran (Liu et al., 2014; Bell, Stoven & Melathopoulos, 2020; Shang et al., 2020). Characterized by an upright trunk, it features glossy green, opposite leaves that are elliptic-ovate to ovate-lanceolate, measuring between 2 to 11 inches in length and 1 to 5 inches in width (Bell, Stoven & Melathopoulos, 2020). The leaves emerge in spring following fragrant winter blooms and undergo a transformation to yellow hues during the fall season. Wintersweet, distinguished by its unique and fragrant flowers, blooms from February to March, providing a vibrant burst of color and fragrance during the winter months (Shang et al., 2020). These strongly scented flowers, delicately hanging on bare stems, typically showcase petals ranging from sulfur-yellow to pale-yellow, often with a purplish-brown center.

The flowers, measuring 1 to 1.5 inches across, contribute to the shrub’s ornamental beauty (Bell, Stoven & Melathopoulos, 2020). Thriving in conditions of full sun to part shade, wintersweet requires moderate watering and is often recommended for hedge use due to its manageable maintenance level and impressive fall foliage (Bell, Stoven & Melathopoulos, 2020). Wintersweet, beyond its ornamental appeal, is acknowledged for its valuable blooms during dormancy.. Cultivars like *Chimonanthus praecox* ‘Grandiflorus’ and *Chimonanthus praecox* ‘Luteus’ have received accolades, including the prestigious Royal Horticultural Society’s Garden Achievement Award (Bell, Stoven & Melathopoulos, 2020). Overall, *Chimonanthus praecox* stands out not only for its aesthetic qualities but also for its adaptability and contributions to garden landscapes (Shang et al., 2020). As of July 2020, the vegetation of China recognizes several distinct species within the *Chimonanthus* genus, each contributing to the rich diversity of this botanical group. These species include *Chimonanthus grammatus* M.C. Liu, *Chimonanthus zhejiangensis* M.C. Liu *Chimonanthus nitens* Oliv., *Chimonanthus praecox* (L.) Link, *Chimonanthus campanulatus* R.H. Chang & C.S. Ding, and *Chimonanthus salicifolius* S.Y. Hu (Roh-Hwei & Chen-Sen, 1980; Liu, 1984). The acknowledgment of these species by the Flora of China holds substantial importance for botanical research, conservation initiatives, and the broader comprehension of plant biodiversity in the region (Jamal et al., 2021).

Historically, taxonomic studies of the Chimonanthus genus have predominantly depended on morphological characters (Dai et al., 2012). However, relying solely on morphology for describing and delineating this genus presents challenges, as these characters can be influenced by environmental factors and may vary across different physiological phases of plants (Wang et al., 2014). Past morphological studies have suggested varying numbers of *Chimonanthus* species, ranging from three to six, and even up to nine based on different morphological evidence (Fan et al., 2010; Dai et al., 2012). However, a recent study utilizing nuclear and chloroplast sequences supported the cataloguing of six species in this genus (Zhou, Renner & Wen, 2006). Two *Chimonanthus* species, *Ch. salicifolius* and *Ch. praecox*, have particularly long histories of utilization and cultivation in China (Dai et al., 2012). *Ch. praecox* iswidely cultivated for its decorative value, especially its sweetly fragrant flowers, *Ch. praecox* holds additional medicinal significance, with its leaves, roots, flowers, and seeds being utilized for medicinal purposes (Lv et al., 2012). The inclusion of molecular data in taxonomic studies enhances the understanding of the *Chimonanthus* genus, contributing to more accurate classifications and aiding in the conservation and sustainable utilization of these plant species.

### Distribution and habitat

*Chimonanthus praecox* has its roots in China, where it is referred to as La Mei Hua and has been cultivated for more than 1,000 years. Its native range spans from the provinces of Hubei and eastern Sichuan to Zhejiang (Zhang et al., 2009; Lv et al., 2012; Huang et al., 2021). The plant reached Japan in the 17th century and became known as Japanese allspice, despite having no practical relation to *Jamaican allspice*, *Pimenta dioica*. The introduction of wintersweet to Britain in 1766 marked its dissemination throughout the western hemisphere, with Lord Coventry contributing to its distribution (Zhang et al., 2009). The genus name, *Chimonanthus*, originates from Greek, signifying ‘winter flower,’ while the species name, praecox, is derived from Latin, meaning early or precocious (Lv et al., 2012).

In terms of cultivation, wintersweet thrives in full sun with moist soil, commencing its flowering in late December and potentially extending into March (Wang et al., 2014). Hardy to the low end of zone 7 (0 degrees), it may experience later flowering and risk frost damage near freezing temperatures (Shang et al., 2020). The deciduous nature of wintersweet allows its flowers to stand out on naked stems, although it has a peculiar habit of retaining around 3% of its leaves, adding a touch of oddness (Bell, Stoven & Melathopoulos, 2020).

Wintersweet responds well to aggressive spring renovation, generating numerous new stems, but it may exhibit sporadic flowering for several years due to its tendency to bloom on the previous year’s wood (McCann, 2022). Interestingly, a plant grown from seed may take up to 14 years to bloom for the first time, and there’s a peculiar behavior where it may not flower if growing vigorously (Wang et al., 2014). This nuanced nature adds to the allure and intrigue of cultivating *Chimonanthus praecox*, making it a plant with both ornamental and historical significance (Wang et al., 2014).

### Morphological features

**Figure 2:**
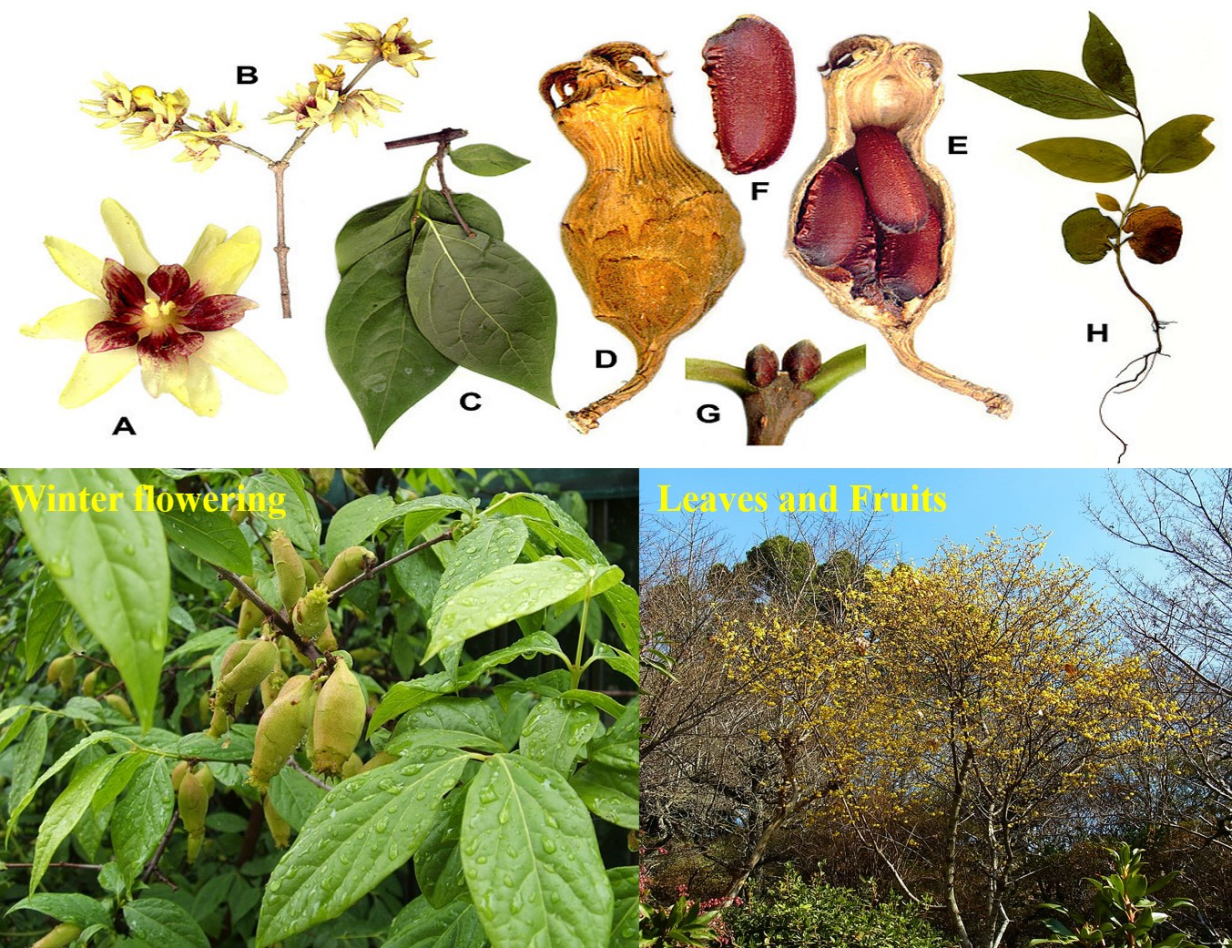
Morphological features of *Chimonanthus praecox* ((A) and (B) flowers; (C) foliage; (D) hypanthium; (E) longitudinal section of hypanthium; (F) fruit; (G) terminal leaf buds; (H) seedling). The picture is taken from https://en.wikipedia.org/wiki/Chimonanthus_praecox.

*Chimonanthus praecox*, a woody shrub, presents a distinctive and versatile profile in the landscape. With an arching, multi-stemmed, and multi-trunked structure, it attains a height of 10 to 13 ft and spans a width of 8 to 15 ft (Liu et al., 2014). The non-showy fruit of this shrub is characterized by an urn-shaped receptacle bearing 5-8 bean-shaped achenes, making it non-ornamental (Kang et al., 2022). Thriving in dappled sunlight or full sun conditions, *Chimonanthus praecox* prefers well-drained, moist soils of varying textures, including clay, loam, and sand. With a slow growth rate and a coarse texture, it is well-suited for coastal and piedmont regions (Kang et al., 2022). Beyond its ornamental value, this shrub holds ecological significance by attracting wildlife and providing edible fruit, contributing to the diversity and vitality of landscapes (Lin & Chen, 2020). The leaves of Chimonanthus praecox are opposite, simple, lustrous, and dark green, ranging in length from 2.5 to 6 inches. The veins, lighter in color, are easily distinguishable. These ovate-lanceolate leaves feature an entire margin, acuminate apex, and cuneate base, presenting a rough texture. As autumn approaches, the leaves undergo a transition to a yellow-green color, as depicted in Fig. 2 (Bell, Stoven & Melathopoulos, 2020).

*Chimonanthus praecox* boasts waxy, cupped flowers, measuring 0.7 to 1 inch, occurring in winter to early spring on leafless branches. Adorned and fragrant with purple centers, these flowers emerge on the preceding season’s growth. The inner tepals are purple or brown, while the outer tepals are yellow (Meng et al., 2021). The stem is squarish, covered with orange-brown and shiny gray-brown lenticels, exhibiting a cane-like development habit (Meng et al., 2021). This versatile shrub finds a place in various landscape themes, including children’s gardens, cottage gardens, and recreational play areas (Lin & Chen, 2020). Its fragrant blooms make it an excellent choice for sensory gardens, especially those designed for the visually impaired. Moreover, *Chimonanthus praecox* attracts pollinators and songbirds, adding ecological value to pollinator and winter gardens (Pichersky & Gershenzon, 2002). Adaptable and engaging, this deciduous shrub seamlessly integrates into diverse landscapes, enriching outdoor spaces with both aesthetic beauty and biodiversity (Lin & Chen, 2020).

## Flower Development and Morphology

### Floral structure

The flowers of *Chimonanthus praecox* stand out as its most distinctive feature, showcasing a bell-shaped structure with a diameter ranging from 2 to 3 cm. This floral marvel exhibits a unique composition, characterized by numerous yellow inner petals and a few outer tepals with a distinctive dull, purplish-brown hue (Zhu et al., 2024). The waxy nature of the tepals adds to the overall appeal of wintersweet flowers. Notably, the flower’s color is predominantly yellow, with inner tepals displaying a captivating range from yellow to red, making it an ideal subject for studying floral color formation in ornamental shrubs (Yang et al., 2018).

### Ontogeny of flowers

The ontogeny of flowers in *Chimonanthus praecox* unfolds in a distinctive and intriguing pattern (Shang et al., 2020). The flowering process is marked by intricate stages, from initiation to full bloom (Kamran et al., 2023). Notably, flower initiation in *Chimonanthus praecox* takes place in spring, yet the blooms grace the plant during the winter months, typically from late December to January. This unique timing is particularly remarkable as the flowering period is synchronized with seasonal climate changes, primarily responding to temperature fluctuations (Kamran et al., 2023). Throughout the summer and autumn, flower buds undergo a slow growth phase, completing the specification, differentiation, and maturation of floral organs, following a pattern observed in many long-lived trees (Kubitzki, 1993). One of the exceptional features of wintersweet is its growth cessation and dormancy establishment before the advent of winter, a common characteristic shared with many long-lived trees. Unlike most flowering trees, wintersweet’s floral buds break, and flowers bloom in midwinter, showcasing an extraordinary adaptation for enduring cold conditions. This unique flowering season requires flowers to possess strong cold hardiness, contributing to the shrub’s ability to thrive in winter climates (Bell, Stoven & Melathopoulos, 2020).

Furthermore, wintersweet flowers exhibit an entirely petaloid perianth without differentiation of sepals and petals (Kamran et al., 2023). This distinctive characteristic not only adds to the ornamental appeal of the plant but also provides researchers with a unique system for elucidating various aspects of flowering-time regulation, flower development, floral bud dormancy, and bud break (Shang et al., 2020). The ontogeny of wintersweet flowers provides a fascinating glimpse into the adaptive strategies of this ornamental shrub, offering valuable insights for both scientific understanding and horticultural applications.

### Factors influencing flower color and fragrance

The floral color and scent profiles of *Chimonanthus praecox* plants exhibit important variations among species and geographical locations, influenced by multiple factors, as outlined by Li et al., (2022) and Cho, Yoon & An (2017). These profiles are subject to fluctuations based on developmental stages, types of tissues sampled, and the season of collection. The intricate interplay of genetic, environmental, and physiological factors contributes to the unique characteristics of wintersweet flowers.

Genetic factors play a pivotal role in determining flower color and fragrance in *Chimonanthus praecox*. Specific genes control pigment synthesis and the production of volatile compounds responsible for fragrance (Kamran et al., 2023). The unique blooming season of wintersweet suggests distinct molecular mechanisms governing flower development compared to spring-blooming plants, highlighting the importance of understanding the genetic makeup (Zhang et al., 2011; Liu et al., 2014). Genetic variations among different cultivars result in the diverse range of colors and scents observed in *Chimonanthus praecox* (Shen et al., 2021; Kamran et al., 2023).

Environmental factors, such as temperature, light intensity, and soil composition, considerably effect flower characteristics (Islam et al., 2021). Wintersweet has progressed to coordinate flowering with seasonal climate changes, especially temperature variations, showcasing the plant’s adaptation to its surroundings (Li et al., 2020). The unusual flowering season, requiring strong cold hardiness, contributes to the distinctive features of wintersweet flowers (Shang et al., 2020). Flower initiation takes place during spring, a common occurrence among many plants. However, the distinctive aspect lies in the fact that the flowers bloom during the winter months, typically appearing late in December or early January. Throughout the summer and autumn, the flower buds undergo a remarkably slow growth phase, where they meticulously complete the processes of floral organ specification, differentiation, and maturation. The extended developmental period of wintersweet, setting the stage for its exceptional flowering season, is marked by the preparation for winter. This involves a phase inherent in the life cycle of long-lived trees, characterized by growth cessation and the establishment of dormancy—a strategic adaptation to overcome the challenges posed by the colder months (Kubitzki, 1993). Unlike the majority of flowering trees, the floral buds of wintersweet break, and the flowers bloom in the midst of winter, creating a captivating display in an otherwise dormant landscape (Shang et al., 2020). This unusual flowering season places unique demands on the flowers, necessitating strong cold hardiness to endure the winter temperatures. Furthermore, wintersweet flowers exhibit a distinctive feature –an entirely petaloid perianth without differentiation of petals and sepals (Liu et al., 2023). This characteristic adds to the plant’s allure and offers researchers a special system for delving into the intricate processes of flowering-time regulation, floral bud dormancy, flower development, and bud break, providing valuable insights into the adaptive strategies of this ornamental shrub (Kubitzki, 1993).

Various physiological processes within the plant contribute to flower color and fragrance. Pigment biosynthesis pathways, hormone regulation, and metabolic activities are crucial components (Liu et al., 2014). Hormones like ethylene can influence both flower color and fragrance production, showcasing the intricate balance of physiological processes (Sui et al., 2015; Shang et al., 2020). The stage of flower development also plays a role in determining color and fragrance, with different compounds synthesized and accumulating during specific stages (Lv et al., 2023). The evolution of flower color and fragrance in *Chimonanthus praecox* is likely influenced by the plant’s interaction with pollinators. (Pichersky & Gershenzon, 2002; Shen et al., 2021). The specific color and scent profile may have evolved to attract certain pollinators, contributing to successful reproduction and ensuring the plant’s survival, aligning with principles of co-evolution (Li et al., 2022). Understanding these complicated interactions and the temporal aspects of physiological processes is crucial for unraveling the factors influencing flower traits in wintersweet.

## Chemical composition of flower fragrance

Traditional and modern methods are employed for the analysis and extraction of volatile organic compounds (VOCs) from plants, with each approach offering unique advantages. Traditional methods include steam distillation and solvent extraction, while modern techniques encompass headspace-solid phase micro extraction (HS-SPME), microwave-assisted extraction and enzyme-assisted extraction, (Madani, Mazouni & Nedjhioui, 2021). A well-established modern method for analyzing flower fragrance, particularly in the context of wintersweet flowers, involves the combination of HS-SPME and gas chromatography-mass spectrometry (GC-MS) (Díaz-Maroto et al., 2008; Feng et al., 2022). This analytical approach allows for the precise quantification and identification of volatile compounds present in the flowers.

Wintersweet flowers are renowned for their intense fragrance, primarily attributed to a combination of volatile benzenoids and terpenoids (including sesquiterpenes and monoterpenes), emitted from nectarines dispersed on the adaxial surface of inner petals (Tian et al., 2019). The key floral fragrance compounds of *Chimonanthus praecox*, including trans-β-ocimene, benzyl acetate, linalool, caryophyllene, and β-myrcene (Zhou, Xiang & Chen, 2007); benzyl acetate, alloocimene, and methyl salicylate (Zhou & Ni, 2010); benzyl acetate, linalool, and methyl salicylate (Yu, 2013); benzyl alcohol, linalool, and methyl salicylate (Li, 2015); and z-muurolene, elemol, z-elemene, L-bornyl acetate, and β-cubebene, (Li et al., 2008), respectively. Understanding these compounds contributes not only to the appreciation of wintersweet’s olfactory charm but also to potential applications in various industries.

The investigation of floral fragrance compounds in *Chimonanthus praecox* from various regions has provided valuable insights into the diverse aromatic profiles of this species. Meng et al. (2021) identified 31 floral fragrance compounds in *Chimonanthus praecox* with dissimilar floral colors in Yunnan, China (Table 1). The main compounds included α-ocimene, eugenol, benzyl acetate, benzyl alcohol, and indole, representing a variety of chemical classes such as phenols, alcohols, terpenes, esters, and heterocyclic compounds. Similarly, studies in different regions revealed varying compositions of floral scent compounds. Li et al. (2022) identified a substantial number of compounds in *Chimonanthus praecox* from Yunnan, whereas 48, 86, 72, 15, 31, 31, 71, 65 and 33 compounds were identified in *Chimonanthus praecox* from Chongqing (Zhengguo et al., 2008), Hubei (Li, 2015), Jiangxi (Xu et al., 2006), Japan (Azuma, Totota & Asakawa, 2005), Henan (Zheng et al., 1990), Shanghai (Deng, Song & Hu, 2004) Zhejiang (Li et al., 2009), Shandong (Li et al., 2008), and Turkey (Özderin, Tiğli Kaytanlioğlu & Fakir, 2021) respectively. These compounds belonged to categories such as alcohols, acids, esters, terpenes, aldehydes, aromatic compounds, and ketones, with alcohols being the most predominant. In the floral scent of *Chimonanthus praecox*, compounds like p-xylene, m-xylene, 2-norbornanemethanol, cyclohepta-1,3,5-triene, o-xylene, germacrene D, 3,4-dimethoxycinnamic acid, and ethylbenzene were identified, contributing to the overall aromatic profile (Meng et al., 2021). The analysis of volatile organic compounds (VOCs) in *Chimonanthus praecox* flowers revealed three prospective biomarker compounds (n-cetane, n-pentadecane, and n-heptadecane) in both excised and living flowers (Deng, Song & Hu, 2004). These findings showcase the chemical diversity of floral fragrances in *Chimonanthus praecox*, influenced by geographical locations and environmental conditions.

**Table 1:** Floral fragrance compounds of *Chimonanthus praecox* (taken from Meng et al., 2021).

**Sr. No.**	**Classification**	**Compound name**
1	Terpenes	Cyclooctatetraene
2		α-Thujene
3		α-Pinene
4		Sabinene
5		α-Phellandrene
6		β-Ocimene
7		α-Ocimene
8		trans-β-Ocimene
9		γ-Terpinene
10		Terpinolene
11		Alloocimene
12		Isoledene
13		β-Caryophyllene
14		Aromandendrene
15		Germacrene D
16		Valencene
17	Alcohols	Benzyl alcohol
18		Cinnamyl alcohol
19	Esters	Benzyl acetate
20		Methyl salicylate
21		Bornyl acetate
22		Methyl cinnamate
23		Cinnamyl acetate
24	Phenols	Eugenol
25	Aldehydes	Benzyaldehyde
26		Cinnamal dehyde
27	Aromatic	p-Xylene
28	Hydrocarbons	o-Cymene
29		m-Cymene
30	Heterocyclic	Indole
31	Others	Phenyl-pentamethyl-disiloxane

## Genetic basis of flower color and fragrance

The *Chimonanthus praecox*, 2n = 22, is renowned for its aesthetic value, particularly its visual (color) and olfactory (scent) traits in its winter-blooming flowers (Jiang et al., 2023). The plant exhibits a diverse range of varieties classified into three groups based on tepal color variation: Concolor Wintersweet (*Chimonanthus praecox* var. *concolor*): This variety is characterized by yellow middle and inner tepals. Patens Wintersweet (*Chimonanthus praecox* var. *grandiflorus*): Featuring yellow middle and red inner petals, this variety adds a touch of vibrancy to the tepal color. Rubrum Wintersweet (*Chimonanthus praecox* var. *intermedius*): The distinctive trait of this variety is the presence of red middle and inner petals, contributing to a rich and varied color palette (Jiang et al., 2023). These classifications based on tepal color highlight the diversity within *Chimonanthus praecox* varieties, providing a spectrum of visual appeal. Such variations contribute to the plant’s popularity in ornamental settings, making it a sought-after choice for those seeking both visual and olfactory delights in winter gardens (Shen et al., 2021).

The study conducted by Shang et al. (2020) aimed to sequence the genome of wintersweet (*Chimonanthus praecox*). The DNA used for genome sequencing was obtained from an accession planted in the campus of Huazhong Agricultural University. The researchers utilized PacBio long reads for sequencing, generating a total of 76.96 gigabases (Gb) of data. This data represented approximately 98.83-fold high-quality sequence coverage of the wintersweet genome, which was estimated to be 778.71 megabases (Mb) in size based on k-mer frequency analysis. Flow cytometry was employed to estimate the haploid genome size of wintersweet, and the result was found to be 805.88 Mb, consistent with the estimation derived from the k-mer method. Subsequently, the generated contigs were subjected to a polishing process using Quiver, resulting in 1,623 contigs with an N50 length of 2.19 Mb. The N50 length is a statistical measure used in genomics to describe the contiguity of a genome assembly, representing the contig length at which half of the total assembly length is contained in contigs of this size or longer. In this case, the N50 length of 2.19 Mb indicates the contiguity achieved in the assembly process, with longer contigs contributing to this measure (Table 2) (Jiang et al., 2023).

**Table 2:** Major indicators of the wintersweet genome (taken from Jiang et al., 2023).

**Assembly feature**	**Statistics**
Estimated genome size (by k-mer analysis) (Mb)	778.71
Scaffold N50 (Mb)	65.35
Contig N50 (Mb)	2.19
Assembled genome size (Mb)	695.36
Longest scaffold (Mb)	85.71
Repeat region % of assembly	47.53
Assembly % of genome	99.42
Average coding sequence length (bp)	1,250
Predicted gene models	23,591
Average exons per gene	5.69

Through a comprehensive genomic study, Shen et al. (2021) have elucidated the molecular foundations of tepal color variation among different wintersweet varieties, with a particular focus on Rubrum wintersweet. The study’s comparative analysis revealed that cyanidin glycosides, present in higher concentrations, critically contribute to the formation of yellow tepals. The key regulatory factor in the biosynthesis of anthocyanins in wintersweet, specifically contributing to the yellow tepal color, is identified as CpMYB1. The mutation of CpMYB1 is implicated in the fading of the red color in the inner tepal of Concolor wintersweet, shedding light on the specific genetic factors influencing the observed variations in tepal color across different wintersweet varieties (Shen et al., 2021). Moreover, beyond the aspect of flower color, the diverse floral scents emitted by different wintersweet varieties underscore the intraspecific variation in the chemical composition and biosynthesis of floral bouquets within the wintersweet genus. This multidimensional approach to understanding both visual and olfactory characteristics enhances the overall appreciation and comprehension of the unique traits exhibited by wintersweet varieties (Lu et al., 2020; Qian et al., 2021; Tian et al., 2020).

The unique blooming season of Wintersweet, occurring in winter rather than the more typical spring flowering of many plants, suggests that it may employ distinct molecular mechanisms in governing flower development (Zhang et al., 2011; Liu et al., 2014). This deviation from the typical flowering season of other plants makes Wintersweet an intriguing subject for studying the genetic and molecular regulation of its flowering process. Furthermore, the variation observed in the floral volatile profiles and pigment compositions among different Wintersweet genotypes adds to its appeal as an ideal target for exploring floral traits in ornamental plants (Yang et al., 2018; Tian et al., 2019). The genome-wide identification of basic helix-loop-helix (bHLH) transcription factors (TFs) is highlighted as a promising avenue for comprehending the biological functions associated with these molecular processes in Wintersweet. However, the current knowledge on the bHLH TF family in Wintersweet is limited, with only a few studies focusing on specific members such as CpbHLH1, CpTT8, CpbHLH13, and CpMYC2 (Zhao et al., 2020), CpTT8 (Qian et al., 2021), CpbHLH13, and CpMYC2 (Aslam et al., 2020). Further research in this direction may provide valuable insights into the regulatory networks governing key aspects of Wintersweet’s unique floral traits.

The recent advancements in genetic research, particularly in flavonoid biosynthesis, have been notable. Researchers have made rapid progress in understanding the mechanisms of pigment biosynthesis, and this includes a focus on flavonoid biosynthesis. Transcriptome and proteome analyses have proven to be valuable tools in elucidating the color biosynthesis mechanisms of various plants, and these methods have been successfully applied in studying commercial plants (Wu, Northen & Ding, 2023). The application of RNA-Seq (transcriptome analysis) and proteomics has been instrumental in uncovering the networks that influence flower color. These techniques enable researchers to identify genes potentially involved in wintersweet flower color biosynthesis. By analyzing the transcriptome and proteome, researchers gain insights into the molecular processes and key players responsible for the distinctive colors and scents observed in wintersweet flowers (Yang et al., 2018).

## Environmental factors impacting flower traits

*Chimonanthus praecox* exhibits a flowering period that varies across different climate zones. The specific timing of flowering can be influenced by regional climate conditions (Xu & Du, 2009). In Northern China, the flowering period typically occurs from February to March, with variations depending on specific locations. For instance, in Beijing, wintersweet starts flowering in late February (Li, Chen & Li, 2012). In Anyang Wintersweet Garden, located in Henan Province, the earliest blooming time is in mid-December (Li et al., 2022), In Sichuan Province, wintersweet enters its initial flowering period in early December (Song, Yuan & He, 2012). Kunming, situated on the Yunnan-Guizhou Plateau in Southwest China, experiences high altitudes, low summer temperatures, and high winter temperatures. In Kunming, wintersweet plants tend to bloom earlier than in other northern regions, usually at the end of November (Li, Yang & Chen, 2022). Wintersweet thrives in cold environments and tends to bloom during low-temperature seasons with minimal rainfall. (Li, Yang & Chen, 2022). The plant is believed to possess genes related to floral development and adaptations, particularly those responding to environmental stress factors (Sui et al., 2012).

The differentiation of buds in plants is a crucial and intricate morphological process that signifies the transition from vegetative growth to reproductive growth. This process not only influences the flowering period but also determines the number of flowers produced (Zik & Irish, 2003). The initiation of bud differentiation marks the end of vegetative growth and the onset of reproductive growth, playing a pivotal role in the overall reproductive strategy of the plant. The mechanisms underlying bud differentiation are complex, and they are often closely linked to the plant’s flowering mechanism. Environmental conditions, including temperature, light, moisture, and mineral content, are key factors that could substantially impact the process of flower bud differentiation (Ito et al., 2014; Xing et al., 2019; Shenping et al., 2020). Various signaling pathways and molecular processes are regulated in response to these environmental factors. Temperature, in particular, has been identified as a crucial environmental factor influencing flower bud differentiation in many plants (Khodorova & Boitel-Conti, 2013; Distefano et al., 2018). Understanding the environmental cues and molecular processes involved in bud differentiation is essential for elucidating the flowering mechanisms of plants. It provides valuable insights into how external factors influence the reproductive development of plants and helps in optimizing conditions for desired flowering outcomes in horticulture.

## Physiological Processes Influencing Fragrance and Color

*Chimonanthus praecox* is a winter-blossoming plant species that holds substantial ornamental value (Zhao et al., 2007; Xiang, Zhao & Chen, 2010). Several molecular studies and practical explorations have been conducted to unravel key mechanisms related to stress resistance, fragrance biosynthesis, cold hardiness, and floral development in this unique species (Xiang, Zhao & Chen, 2010; Sui et al., 2012; Zhao et al., 2020). One distinctive feature of wintersweet is its unique flower structure, characterized by three layers of scale-like tepals: outer tepals, middle tepals, and inner tepals (Yang et al., 2018). These tepals are waxy and translucent, contributing to the overall visual appeal of the flower. The flowers are drooping on the wintersweet branch, with red tepals typically positioned in the center of the flower. The red inner tepals, however, are often barely visible due to their arrangement (Yang et al., 2018). The primary determinant of the overall color of the wintersweet flower is the middle tepals, usually in shades of yellow or light yellow. In contrast, the color of the inner tepals varies from yellow to red. Over the centuries, breeders of wintersweet have obtained new cultivars with red middle tepals. This emphasis on achieving red middle tepals highlights the aesthetic preferences and goals of cultivators working with wintersweet, aiming to enhance its visual appeal and diversity (Yang et al., 2018). The combination of ornamental value, winter blossoming, and unique floral characteristics makes wintersweet a subject of interest for both researchers and horticulturists, contributing to ongoing efforts in breeding and cultivating new and improved varieties.

## Ecological and Evolutionary Perspectives

Mesangiosperms, encompassing magnoliids, monocots, and eudicots, represent a dominant group, constituting 99.95% of angiosperm species. Among mesangiosperms, magnoliids have been historically regarded as an early lineage and are classified into four orders: Canellales, Laurales, Magnoliales, and Piperales, comprising approximately 9,000 species (Group, 2016). Magnoliids share distinctive morphological features, including branching-veined leaves, pollen with a single pore, and trimerous flowers (Jiang et al., 2023). Recent advancements in genomics, specifically genome sequencing of various magnoliid species such as *Liriodendron chinense*, *Cinnamomum kanehirae*, *Persea americana*, and *Piper nigrum*, have provided valuable insights into the evolutionary relationships among eudicots, monocots, and magnoliids (Rendón-Anaya et al., 2017; Chaw et al., 2019; Hu et al., 2019; Chen et al., 2019). Analysis of 502 low-copy nuclear (LCN) gene sets from *L. chinense*, along with 12 other species, placed magnoliids outside the sister lineage formed by eudicots and monocots (Chen et al., 2019). Similar findings were corroborated by analyses of 176 LCN gene sets from *P. americana* and other 18 flowering plants, as well as 82 LCN gene sets from *P. nigrum* and 20 other land plants (Rendón-Anaya et al., 2017; Hu et al., 2019). However, a contrasting phylogenomic analysis, utilizing 211 LCN gene sets from *C. kanehirae* and 12 other seed plants, suggested that magnoliids might be the sister group to eudicots (Hu et al., 2019).

*Chimonanthus praecox*, belongs to the Calycanthaceae family within the order Laurales, Calycanthaceae is a small and ancient family consisting of ten species distributed among four genera: *Calycanthus* L in North America, *Sinocalycanthus* Cheng & S. Y. Chang, and *Chimonanthus* L. in China, and *Idiospermum* black in Australia. These genera exhibit variations in flower color, flowering time, and geographical distribution, contributing to the overall diversity within the Calycanthaceae family (Kubitzki, 1993; Ye & Bingtao, 2000; Zhou, Renner & Wen, 2006).

Genetic studies related to ecological and evolutionary perspective on *Chimonanthus praecox* have been relatively limited (Zhou, Xiang & Chen, 2007). A study employing amplified fragment length polymorphism (AFLP) and inter simple sequence repeat (ISSR) markers investigated seven natural *Chimonanthus praecox* populations, revealing high genetic diversity and low gene flow among populations, though the population structure was not explored in depth (Zhao et al., 2007). Another study, utilizing sequence-related amplified polymorphism (SRAP) markers on five natural populations, suggested low genetic diversity, high gene flow, and minimal differentiation among populations in central China (Zuo et al., 2009), However, caution is warranted as the limited number of populations may not fully capture the overall genetic relationships across the species’ distribution. In 2007, genetic diversity was assessed in seventy-two wintersweet clones from two Chinese regions using 11 inter simple sequence repeat (ISSR) and 19 random amplified polymorphic DNA (RAPD) markers (Zhou, Zhao & Chen, 2007; Zhao et al., 2007). High genetic differentiation among these clones was observed, although it’s worth noting that all the clones were propagated asexually.

## Conclusion and Future Directions

In conclusion, the exploration of *Chimonanthus praecox*, or wintersweet, has unveiled a fascinating tapestry of genetic, environmental, and physiological factors influencing its distinctive flower color and fragrance. The bell-shaped flowers, ranging from yellow to red, with unique adaptations to winter blooming, make wintersweet a captivating subject for scientific inquiry and landscape applications. The review emphasizes the pivotal role of genetic makeup in determining floral traits. Studies on pigment biosynthesis pathways, hormone regulation, and metabolic activities provide insights into the intricate processes shaping flower color and fragrance. The identification of specific genes governing pigment synthesis, such as quercetin and cyanidin glycosides, sheds light on the molecular basis of tepal color variation. Environmental factors, including light intensity, temperature, and soil composition, considerably influence wintersweet’s flower characteristics. The synchronization of flowering with seasonal climate changes, especially temperature fluctuations, showcases the plant’s adaptive strategies for enduring cold conditions. Physiological processes, including the influence of hormones like ethylene, contribute to the balance of floral characteristics. The review underscores the importance of understanding temporal aspects and developmental stages in unraveling the factors influencing flower traits. The study of floral scent compounds in different varieties across geographical locations further enriches our understanding of wintersweet’s diverse aromatic profiles. Wintersweet’s position as an ideal candidate for exploring floral traits in ornamental plants is underscored, offering opportunities for landscape applications. In essence, this comprehensive review encapsulates the intricate interplay of genes, environmental factors, and physiological processes that contribute to the enchanting flower color and fragrance of *Chimonanthus praecox*. The findings not only deepen our understanding of this winter-blooming beauty but also pave the way for leveraging its ornamental and economic potential in diverse landscapes.

The insights derived from this review on the genetic and environmental factors influencing the flower color and fragrance of *Chimonanthus praecox* offer valuable guidance for landscape architects and horticulturists. By understanding the molecular mechanisms and ecological adaptations of this winter-blooming shrub, landscape designers can strategically incorporate diverse *Chimonanthus praecox* cultivars to enhance visual and olfactory aesthetics globally. The review serves as a foundation for informed plant selection, allowing for the creation of captivating and sustainable landscapes that thrive in various climates, enriching outdoor spaces with the unique beauty and fragrance of wintersweet blooms.

Future research should leverage the ecological importance of *Chimonanthus praecox* for pollinator conservation and ecosystem services. Integrating cutting-edge technologies, like CRISPR-based gene editing, offers novel avenues for tailoring specific floral traits. Harnessing these attributes for innovative landscape designs and urban green spaces can contribute to biodiversity and enhance environmental aesthetics. Addressing challenges and advancing research in these future directions holds promise for unlocking the full potential of *Chimonanthus praecox* in various fields.

## Additional Information and Declarations

### Data Availability

The following information was supplied regarding data availability: This is a literature review.
